# “Googling” for Cancer: An Infodemiological Assessment of Online Search Interests in Australia, Canada, New Zealand, the United Kingdom, and the United States

**DOI:** 10.2196/cancer.5212

**Published:** 2016-05-04

**Authors:** Forough Foroughi, Alfred K-Y Lam, Megan S.C Lim, Nassim Saremi, Alireza Ahmadvand

**Affiliations:** ^1^ Department of Pathology School of Medicine Shahid Beheshti University of Medical Sciences Tehran Islamic Republic Of Iran; ^2^ Cancer Molecular Pathology School of Medicine and Menzies Health Institute Queensland Griffith University Gold Coast Australia; ^3^ Centre for Population Health Burnet Institute Melbourne Australia; ^4^ School of Clinical Sciences Faculty of Health Queensland University of Technology Brisbane Australia

**Keywords:** cancer, neoplasms, infodemiology, epidemiology, geographic mapping, Google Trends, Internet, consumer health information

## Abstract

**Background:**

The infodemiological analysis of queries from search engines to shed light on the status of various noncommunicable diseases has gained increasing popularity in recent years.

**Objective:**

The aim of the study was to determine the international perspective on the distribution of information seeking in Google regarding “cancer” in major English-speaking countries.

**Methods:**

We used Google Trends service to assess people’s interest in searching about “Cancer” classified as “Disease,” from January 2004 to December 2015 in Australia, Canada, New Zealand, the United Kingdom, and the United States. Then, we evaluated top cities and their relative search volumes (SVs) and country-specific “Top searches” and “Rising searches.” We also evaluated the cross-country correlations of SVs for cancer, as well as rank correlations of SVs from 2010 to 2014 with the incidence of cancer in 2012 in the abovementioned countries.

**Results:**

From 2004 to 2015, the United States (relative SV [from 100]: 63), Canada (62), and Australia (61) were the top countries searching for cancer in Google, followed by New Zealand (54) and the United Kingdom (48). There was a consistent seasonality pattern in searching for cancer in the United States, Canada, Australia, and New Zealand. Baltimore (United States), St John’s (Canada), Sydney (Australia), Otaika (New Zealand), and Saint Albans (United Kingdom) had the highest search interest in their corresponding countries. “Breast cancer” was the cancer entity that consistently appeared high in the list of top searches in all 5 countries. The “Rising searches” were “pancreatic cancer” in Canada and “ovarian cancer” in New Zealand. Cross-correlation of SVs was strong between the United States, Canada, and Australia (>.70, *P*<.01).

**Conclusions:**

Cancer maintained its popularity as a search term for people in the United States, Canada, and Australia, comparably higher than New Zealand and the United Kingdom. The increased interest in searching for keywords related to cancer shows the possible effectiveness of awareness campaigns in increasing societal demand for health information on the Web, to be met in community-wide communication or awareness interventions.

## Introduction

The Internet is being used globally by millions of people on a daily basis for finding health information [1]. The analysis of collective trends and patterns in seeking information about health and medical conditions has helped in giving insights into information needs at the population level [2]. It is now more than a decade since the interdisciplinary area of “infodemiology” has emerged [3] and developed to scientifically assess the distribution and determinants of information in electronic media, with main focus on the Internet. Informing public health and public policy has been considered the eventual goal in infodemiological studies [4]. From the demand side of infodemiological approaches, the analysis of queries from search engines to shed light on the status of various diseases and the analysis of people’s health information-seeking behaviors have gained increasing popularity, especially during the last 4-5 years [4]. Many researchers have provided important insights into health-related behavior of populations, specifically for various communicable and noncommunicable diseases [5]. Predicting the future burden of health issues and diseases, improving public health practice, and expanding the potentials of research in health care have shown not only development, but also standardization in recent years [6].

Google Trends (GT) Web service is a unique and popular service available to assess data on people’s interest in Internet search using Google. As a free tool, it has widely been used for infodemiological studies on a variety of communicable and noncommunicable diseases and conditions [7-18]. Nevertheless, published studies in the recent years on infodemiology of chronic diseases, especially cancer, are limited [19] and they are outnumbered by the studies on infectious diseases, mainly influenza [20].

As cancers are among the most common causes of morbidity and mortality [21], it is anticipated that many people search the Web for information regarding various cancers. One of the first infodemiological studies on cancer using Google Insights for Search (former name of GT) was done by Glynn et al who assessed the relationship between breast cancer awareness campaign and Internet search activity from 2004 to 2009. Moreover, they determined the overall levels of online activity regarding breast cancer along with prostate and lung cancers [22]. In addition, Zhang et al assessed Internet search query data, specifically on tobacco and lung cancer, in the United States, Canada, the United Kingdom, Australia, and China from January 2004 to January 2014 using GT. They aimed to conduct seasonality analyses to detect the pattern in seeking information regarding tobacco and lung cancer at the international level [23]. Recently, Bloom et al [24], Murray et al [25], Schootman et al [26], and Rosenkrantz and Prabhu [27] have shown the usage of GT data for studying skin cancer, mouth cancer, cancer screening, and imaging-based cancer screening, respectively.

However, these recent studies have shortcomings in terms of giving a bigger picture on the global health information-seeking patterns of people regarding cancer as a major noncommunicable disease entity. Almost all of them are done in the context of one country (ie, the United States; except for the work by Zhang et al [23]), so they fall behind in terms of supporting public health practice or policy changes in other countries. Additionally, not only have they characteristically focused on one cancer type, but also they have partially assessed “parts” of the cancer diagnosis and care continuum (eg, screening, risk factors, or awareness).

Therefore, infodemiological assessment of one cancer type, in one country, from a noncomprehensive point of view, brings the opportunity for the development of studies with more comprehensive approaches. The objectives of our infodemiological study are based on the possibilities provided by GT for comparing the “Cancer” keyword classified as “Disease,” and its related keywords, simultaneously across 5 geographic locations, from January 2004 onward. For bridging the gaps in previous studies, we aimed to do the following:

1. Provide an international picture on health information–seeking behavior of people on “cancer” in the past 12 years, using search query data;

2. Assess the most popular types of cancers that have shown search interest by people in various countries;

3. Uncover the various keywords and subjects searched by people in relation to cancer;

4. Determine the degree of correlation between the main indexes of cancer burden and interest in searching for cancer;

5. Reveal whether there is any correlation between people’s search interests in various countries.

## Methods

For this infodemiological study, we used GT (Google Inc, Mountain View, CA, USA; Last Accessed on February 6, 2016) to assess demand-side data on people’s interest in Internet search using Google for “Cancer,” classified by the search engine as “Disease.”

### Google Trends Methodology

Google Trends analyzes a fraction of the total Google Web searches over a period of time on a daily basis, extrapolates the data to estimate the search volume (SV), and displays SV index graphs. Comparisons between search terms or geographical areas are possible over time since January 2004. Such terms must reach a threshold of traffic to appear in the results. To control for artificial effects of repeated queries over a short period of time from a single user, this kind of repeated queries are removed automatically [26].

Google Trends analyzes number of searches over time in Google.com for a specified term relative to the total number of searches. This proportion known as “Search Volume Index” shows “the likelihood of a random user to search for a particular term from a certain location at a certain time” [28]. Because of the relativity (search for a specific keyword divided by the total number of searches), SV has the display scale of 0 to 100. Differences in the population of Internet searchers in various regions should be accounted for. Therefore, GT has a normalization process to justify the total SV in a region in a given time period, to not automatically give the highest rankings to those regions with the most SVs, and to make datasets from different regions or cities comparable with each other. For this to happen, GT divides a set of search data from a region by the total traffic from the same region to cancel out the effects of differences in the population of searchers and the number of search hits. After the completion of normalization, each SV point is divided by the highest SV and multiplied by 100 to be shown as percentages on the graphs. Thus, regions that have gained higher or lower normalized values during the time frame will be correspondingly close to 100 or 0, respectively. The same process is used for determining top cities. A downward SV trend in graphs does not correspond necessarily to a decrease in absolute traffic for a search term; it just shows that its popularity is decreasing [26].

Google Trends classifies important search terms as meaningful entities; for example, in this case, on typing “cancer” in GT search bar, it shows the classification of this search term as “Disease.” Google Trends also automatically categorizes the terms under prespecified categories (in this case, “Health”) and represents ranks of the search categorized under particular categories. Categories with higher ranks are shown first. Significant growth of searching for a term in a given time period in comparison with the preceding time period will highlight that term as “Rising searches” and show its increasing popularity. The term “Breakout” instead of actual percentages in “Rising searches” means a change in popularity of a search term of more than 5000% [26]. This term is specific to Google and the 5000% percentage is not affected by market share of Google in the search engine market.

Google Trends has been active since 2004 using the abovementioned methodology in background. Google introduced another complimentary service named “Google Insights for Search” in 2008 with advanced visualizations for businesses. This service was merged into GT in 2012, and therefore GT has improved in terms of visualization since then [29].

### Preliminary Keyword Searching and Adjusting Google Trends Parameters

The default time span was from January 2004 (as baseline) to the end of December 2015. Preliminary searching was initiated using the term *cancer*. Then, GT created a graph showing global trends in interest over time for this keyword in more than 50 countries during our defined period. It had a vertical scale range from 0 to 100 in which the number 100 represented the peak SV. The term *cancer* is used mainly as a keyword in the English language; although, the term is similar in French, Swedish, and Romanian languages. However, for adding cross-country comparisons and international perspective as well as assessing related keywords, we decided to focus on English-speaking countries to be able to evaluate keywords of interest related to cancer.

It was shown that “Cancer” had been automatically classified by GT under these 3 categories: Health, People & Society, and Arts & Entertainment. Because GT recommends the most relevant and popular category in the first rank (ie, “Health”), we limited all of our next searches to Health category by selecting “Health” in the “Category” drop-down menu.

### Assessing the Geographical Distribution of Search Interests and Rising Keywords

In the next step, for the international perspective of our study and cross-country comparisons, we extracted top regions (ie, countries) and cities in the “Regional interest” section. Therefore, based on the results from regional interests, we selected top English-speaking countries in descending order of corresponding averages of their weekly SVs (ie, the United States, Canada, Australia, New Zealand, and the United Kingdom). Google Trends adjusts for different population sizes in various countries and cities in calculating SVs. We extracted the “Top” Queries in each country and documented their SVs. This provided us with quantitative index for popularity of a search term from the user’s perspective. Then, we selected “Rising” Queries related to cancer in all 5 countries separately. This showed the quantity of progression in popularity of search from 2004 to 2015 for cancer-related terms.

### Data Handling and Statistical Analysis

At every stage, we exported data from GT as a comma-separated values (CSV) file into Microsoft Office Excel 2010 (Microsoft, Redmond, WA, USA) for cross-checking, description, and refinement for graphing. Graphs and maps were extracted using real-time screen snapshots from the GT website.

Moreover, to examine consistency of trend data between countries and analyze linear and temporal patterns of seasonal components among countries and their possible associations, we calculated the pairwise cross-correlations of these SVs to show the direction and degree of changes in SVs in one country in accordance with changes in SVs in another. Logically, the correlation analysis quantifies the degree of correlation between these seasonal components and shows us the time-shifts among different countries regarding seasonality of the searches about cancer. High cross-correlations between countries mean common temporal patterns in information-seeking behavior that can be used in selecting the appropriate timing of international campaigns [23].

We also assessed correlations between the rank of these 5 countries in terms of the incidence of cancer in 2012 and their corresponding ranks in the average SV for each country between 2010 and 2014. This was done separately for each year using Spearman rank correlation. Data for the incidence of cancer were based on Ferlay et al [30]; for men and women combined, age-standardized incidence rates for all cancers (excluding nonmelanoma skin cancer) per 100,000 were as follows: Australia (323.0, world rank: 3), the United States (318.0, world rank: 6), Canada (295.7, world rank: 12), New Zealand (295.0, world rank: 13), and the United Kingdom (272.9, world rank 23).

The SPSS version 22.0 (IBM Incorporated, New York, USA) was used for all statistical analyses.

## Results

Google Trends recorded 626 SV-weeks for each of the 5 countries from January 2004 to December 2015.

**Figure 1:**
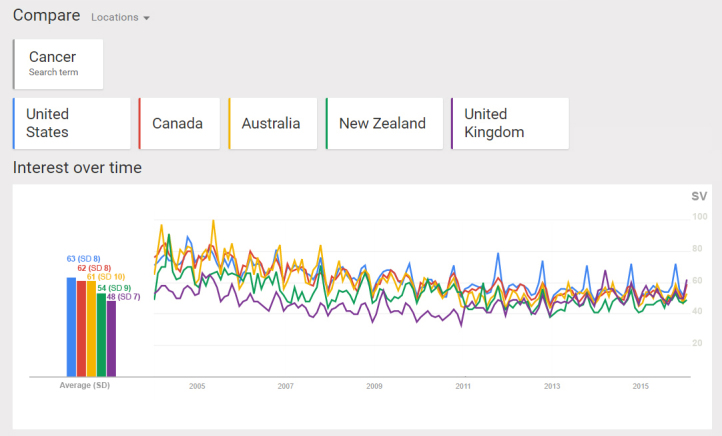
Average interest over time in searching for Cancer as a Disease in Google (shown as search volume [SV] on a scale of 0-100) in the United States, Canada, Australia, New Zealand, and the United Kingdom; January 2004 to December 2015; higher numbers mean higher interest.

The average SV was highest in the United States, 63 (SD 8), and lowest in the United Kingdom, 48 (SD 7). The overall pattern showed a slight decrease in searching for cancer from 2004 to 2011 and then a small increase in the later years. There were also patterns of spikes in SVs, in nearly all countries, more noticeable in the United States, mostly during and around October each year. Apart from these regular spikes, there were two noticeable spikes in SVs in Australia in May 2005 and in the United Kingdom in March 2014, for which GT could not identify possible reasons (Figure 1). Thus, based on further information provided in the Guinness World Records, we found that Cancer Council Australia held the largest tea party in May 2005 for charity fundraising involving 280,246 participants at 6062 locations across the country [31]. Moreover, the United Kingdom’s National Health Service conducted a very large “Be Clear on Cancer” symptom awareness campaign between February and March 2014 [32].

**Figure 2:**
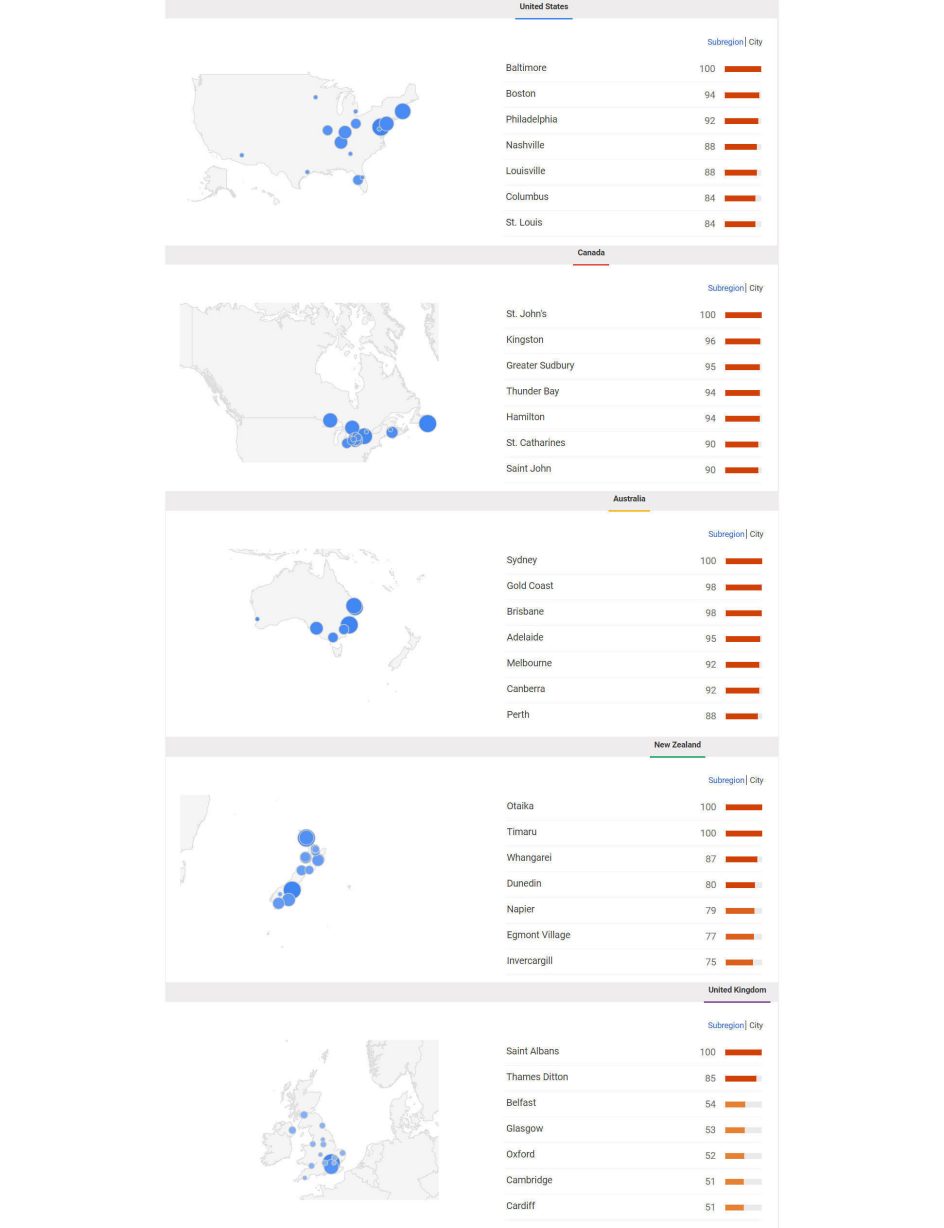
Geographic distribution of searching for cancer in Google in the United States, Canada, Australia, New Zealand, and the United Kingdom; top cities and their corresponding search volumes; January 2004 to December 2015. The scale is up to 100; higher numbers (shown as larger circles) mean higher interest in searching, independently in each country.

Figure 2 shows the specific geographic distribution of searching for cancer by city, independently in each country. Baltimore (United States), St. John’s (Canada), Sydney (Australia), Otaika (New Zealand), and Saint Albans (United Kingdom) were the top cities searching for cancer in their corresponding countries (SV=100). Patterns of geographic clustering were more noticeable in the United States and Canada.

Tables 1 and 2 summarize the “Top searches” and “Rising searches” in Google and their corresponding SVs and growth percentages, respectively.

**Table 1:** “Top searches” in Google related to *cancer* and their corresponding search volumes in the United States, Canada, Australia, New Zealand, and the United Kingdom; January 2004 to December 2014.

Top queries		Search volume
United States		
	Breast cancer	100
	Breast	100
	Cancer symptoms	75
	Lung cancer	40
	Colon cancer	30
Canada		
	Cancer symptoms	100
	Breast cancer	90
	Lung cancer	45
	Prostate cancer	40
	Prostate	40
Australia		
	Cancer symptoms	100
	Breast cancer	90
	Skin cancer	70
	Cancer council	45
	Lung cancer	40
New Zealand		
	Breast cancer	100
	Cancer symptoms	100
	Symptoms	100
	Bowel cancer	50
	Prostate	45
United Kingdom		
	Cancer symptoms	100
	Breast cancer	75
	Symptoms of cancer	40
	Bowel cancer	35
	Lung cancer	35

**Table 2:** “Rising searches” in Google related to *cancer* and their corresponding growth percentages in the United States, Canada, Australia, New Zealand, and the United Kingdom; January 2004 to December 2014.

Rising queries		Growth percentage^a^
United States		
	Stage 4 cancer	250%
	Signs of cancer	170%
	What is cancer	140%
	Symptoms of cancer	90%
	Cancer symptoms	80%
Canada		
	Stage 4 cancer	350%
	Signs of cancer	100%
	Cancer cure	90%
	Pancreatic cancer	90%
	Ovarian cancer symptoms	80%
Australia		
	Skin cancer clinic	450%
	Symptoms ovarian cancer	400%
	Bowel cancer symptoms	200%
	Symptoms of cancer	200%
	Cancer symptoms	180%
New Zealand		
	Breast cancer NZ^b^	>5000%
	Ovarian cancer	>5000%
	Stomach cancer	>5000%
	Symptoms bowel cancer	>5000%
	Symptoms of cancer	>5000%
United Kingdom		
	Cervical cancer symptoms	400%
	Ovarian cancer symptoms	300%
	Signs of cancer	300%
	Pancreatic cancer	190%
	Symptoms of cancer	180%

^a^Google Trends classifies terms with over 5000% increase as “breakout” and does not give exact figures.

^b^NZ: New Zealand.

“Breast cancer” was consistently the first- or second-ranked diagnostic entity appearing in the top 5 searches related to cancer, with SVs ranging between 75 and 100. It was followed by “lung cancer” in the United States and Canada, “skin cancer” in Australia, and “bowel cancer” in New Zealand and the United Kingdom, with SVs of roughly half of that of breast cancer.

The top 5 rising search terms related to cancer in New Zealand between 2004 and 2015 showed a breakout growth percentage in their search interests, that is, over 5000% increase, whereas the rising searches in other 4 countries experienced a fairly smooth growth over the same time period. “Pancreatic cancer” (Canada, United Kingdom) and “ovarian cancer” (New Zealand) were the types of cancer showing greatest increase in search interest between 2004 and 2015.

Table 3 demonstrates the results of testing for cross-correlation between seasonal components of searching for cancer during the time period between the 5 countries.

**Table 3:** Pairwise Pearson correlation coefficients for weekly SVs between the United States, Canada, Australia, New Zealand, and the United Kingdom; January 2004 to December 2014 (all correlation coefficients were significant at the .01 level [2-tailed]).

	Canada	Australia	New Zealand	United Kingdom
United States	.86^a^	.77^a^	.56^b^	.42^b^
Canada		.79^a^	.53^b^	.40^b^
Australia			.58^b^	.42^b^
New Zealand				.22^c^

^a^Strong correlation.

^b^Moderate correlation.

^c^Weak correlation.

The highest coefficient was seen between the United States and Canada, whereas the weakest was between New Zealand and the United Kingdom and the pattern was consistent in various years. Cross-correlation was strong between the United States, Canada, and Australia (>.70). Correlation was moderate between these 3 countries with New Zealand and United Kingdom. Nevertheless, all correlation coefficients were positive and statistically significant.

For these 5 countries, the highest Spearman rank correlation coefficient was between the incidence of cancer in 2012 and the average SV in the year after (ie, 2013; ρ=.8); although, the *P* value was not statistically significant (*P*=.104).

## Discussion

It seems that people’s interest in googling *cancer* in the United States, Australia, Canada, New Zealand, and the United Kingdom, as major English-speaking countries, is becoming comparably similar toward 2011-2015. Additionally, the temporal trend in searching about cancer is most strongly correlated between the United States, Canada, and Australia. Seasonal trends demonstrate that people may be in increased need for getting information—and possibly health services or care—regarding cancer, particularly during or near to October. Our findings contribute to infodemiology of cancer with an international perspective [23].

Previous research by Cooper et al has shown that searching activity related to cancer can be associated with estimates of the burden of cancer in three parameters; namely, cancer incidence, cancer mortality, and cancer news coverage. They also evaluated the periodicity of cancer search activity in Yahoo! and showed that estimated incidence and mortality of specific cancers were moderately correlated (rank correlation between .50 and .66; *P*=.015 to *P*=.001) with Yahoo! search activity. The volume of cancer news coverage was highly correlated with Yahoo! cancer search activity, especially on weekdays and during national cancer awareness months. The authors concluded that assessment of health information-seeking behavior using Internet search activity could be utilized as an innovative passive surveillance tool, mainly for assessing and predicting potential disease burden [33]. Although statistically insignificant, the rank correlation coefficient between the incidence of cancer in 2012 and SV in the next year may show an association between search and overall burden of cancer in these areas at ecological levels. This is a promising area of research on this topic that needs more sophisticated statistical techniques (such as time-series or regression analyses) and the replication of our methodology on specific types of cancer for cross-country comparisons.

In this investigation, we were able to show higher SVs in nearly each of the 5 countries in October, corresponding to breast cancer awareness month. This is a fact that has also been shown by Glynn et al. They clearly demonstrated that in each October, online activity levels relating to breast cancer consistently increase, significantly higher in comparison to lung or prostate cancer (*P*<.001). They inferred that the annual breast cancer awareness campaigns, in comparison to other initiatives for cancer awareness, have been more effective as they have hugely accelerated online search activity. Therefore, the lessons learned from the experience of breast cancer awareness months would additionally be useful for other cancers [22]. Our results also correlate well with previous work that examined cancer search activity using the Yahoo! search engine between 2001 and 2003. Breast cancer ranked first of 23 cancers in terms of search activity, ahead of lung cancer in second place and prostate cancer in fifth [33].

Zhang et al [23] have recently demonstrated the moderately high cross-correlations and seasonality of searching for tobacco and lung cancer in the United States, Canada, the United Kingdom, Australia, and China. Similar findings might be accessible by replicating our research methodology on specific types of cancer and their corresponding risk factors. High cross-correlation could also reflect that certain cancer promotion and awareness campaigns that are propagated over the Internet can impact more than one country at the same time, thus increasing the chances of being taken by diverse populations.

The progressive popularity of searching for various types of cancer in different countries—“Pancreatic cancer” (Canada, United Kingdom) and “ovarian cancer” (New Zealand)—is implicitly reflecting the societal demand for specific information on different cancer entities, apart from the burden of known major types of cancer in people’s country of residence. On the basis of GLOBOCAN 2012, in Canada, pancreatic cancer is ranked 13 based on estimated age-standardized incidence in both sexes [34]. In New Zealand, ovarian cancer is the fifth most frequent cause of death from cancer in females by total number of cases. This becomes more important if we consider the fact that there has been debate on disassociation between the incidence of cancer conditions and SVs for related search terms to those conditions. The reason for latter debate has been the potentially large influence on health-related SVs by the recognition or diagnosis of conditions by celebrities and media [35], which may have an effect on public interest in searching. This fact has also been demonstrated by Noar et al [36]; as they showed that for digital surveillance to strengthen cancer control research and practice, one should be aware that in specific cases (pancreatic cancer in their study), diagnosis of or death because of cancer in public figures may stimulate online information seeking related to the disease entity. In addition, Evans et al described the “Angelina Jolie effect” showing the huge or long-lasting effects of media on a health topic hit to generate better understanding in society about diagnostic tests and management options for breast cancer. Angelina Jolie’s decision to undergo risk-reducing mastectomy after being tested positive for the *BRCA1* gene mutation was one of the longest lasting news stories that affected referrals specific to assessment of breast cancer family history, request for *BRCA1/2* testing, and enquiries for risk-reducing mastectomy, especially in the United Kingdom around May 2013 and onward [37].

Moreover, we noticed infrequent peaks in the SVs in Australia (May 2005) and the United Kingdom (March 2014). Although GT itself flags important news and events related to peaks in the SV graphs, it showed nothing related to these 2 visible aberrancies. We could identify 2 events that seemed possibly related to these less-than-usual spikes in searching; Australia holds the world record for the largest national cancer charity fundraising act in May 2005 and the United Kingdom started a large cancer awareness program in February-March 2014. These findings show the important effects of campaigns on raising the demand for information via searching the Internet. Moreover, they highlight the need for additional cross-country comparisons on health-related information searches, as the differences between various countries would have not been shown if there had not been the possibility of comparing the trends together. In addition, by finding the probable explanations in any peak search activity related to health-related keywords, researchers may end up finding effective awareness activities or experiences in a country, which can be used in informing public health promotion and policies.

Seasonal patterns in information seeking concerning cancer widely exist between these 5 countries. The high cross-correlation between the cancer search trends of Australia, Canada, and the United States reflects the fact that these countries may be able to collaborate to start awareness campaigns at the peak of information seeking, because the interest of information seekers in cancer information would be similarly high at intended times [23].

In an analysis of more than 12,000 people in 12 countries, it has been reported that more than 45% of individuals who have searched the Web for health-related information have done so to self-diagnose a condition [38]. There has also been a report showing Internet search for query data are correlated with patients’ visits to physicians’ offices [17]. We found that in each of these 5 countries, “cancer symptoms” was among the top searches and this finding may reflect the fact that people not only want to get more information about the disease itself, but also may become able to check whether they themselves (or anybody related to them) can provisionally be considered to be at risk for cancer.

### Limitations of Our Study

Google Trends may be suited for tracking search behavior in developed countries because it requires large populations of users in order to provide effective estimates. The main reason is that terms that reach a threshold of traffic appear in GT results. Moreover, GT is available in a limited, albeit increasing, number of languages and it does not support all countries or territories at the moment [39]. Because *cancer* and its related terms are mainly English, the usability of results might be limited to major English-speaking countries. Therefore, the generalizability of our findings might be limited because of sampling data. Our research may be reproduced in the future by including other countries in which English is a major language (eg, India, Pakistan, and South Africa) and by assessing the trends in other languages.

Searches for cancer may not be exclusively done using Google. Evidence from gray literature (eg, industry reports, market research results) has consistently shown that Google has been the largest player in the search engine market, having the market share of at least 50% in various developed countries since 2005 [40] and across the time frame of our study (range 50%-85%) [41]. However, other search engines such as Yahoo! or Bing are also being used by people to search for information. Specifically, data from gray literature shows that more than three-fourth of people in the United States start their online health seeking at a general search engine (eg, Google, Bing, or Yahoo!), not on websites that specialize in health information [42]. However, we have not been able to assess the trends of people’s interests in other search engines because of the proprietary availability of their Web services. Moreover, we cannot describe the demographic characteristics of Internet searchers in different countries because there are no data available from GT on demographics. This may limit the true comparison of information needs and the differences seen within and between regions and cities.

We should also highlight the fact that the presence of pharmaceutical companies and research centers in some locations might have affected the SVs in specific cities across these 5 countries. However, as the assessment of clustering needs extra data on covariates related to geographical locations, studying the reasons for clustering has been out of the scope of our research. This can be an important question to be answered in future studies.

Finally, it should be mentioned that although GT classifies “Cancer” as “Disease” and we had chosen “Health” as the major category of this assessment, we cannot assure that GT differentiates or accounts for false cognates or homonymous words in the search patterns or related keywords. The methodological literature on this classification or categorization is not well elaborated and is in need of further clarification by Google itself or future research.

### Conclusions

More dependence on the Internet worldwide, although challenging in some aspects, provides a wealth of information to show the collective thoughts and needs of populations, which can be assessed for their health issues. Exploring increasingly available online data including Internet search queries and social media information can provide novel insights for public health research and promotion. Google Trends, with its potentials, is a convenient and accessible tool to help researchers assess infodemiological aspects of health and medical conditions of interest in their populations [20,39,43].

Our study shows that GT is also a valuable tool to provide us estimates on the interest in high-burden disease entities such as cancer [44,45]. We propose using GT for getting insight into deeper aspects of problems and challenges related to cancer awareness in order to assess the status quo and to determine the need for detailed research projects on specific subjects in areas that have highest need. It may also be of help to policy makers in tailoring cancer awareness programs to areas that need them the most.

## Abbreviations

GT: Google Trends
SV: search volume
